# The sex pheromone heptacosane enhances the mating competitiveness of sterile *Aedes aegypti* males

**DOI:** 10.1186/s13071-023-05711-6

**Published:** 2023-03-15

**Authors:** Lin-Min Wang, Ni Li, Mao Zhang, Qi Tang, Hong-Zheng Lu, Qing-Ya Zhou, Jia-Xuan Niu, Liang Xiao, Zhe-Yu Peng, Chao Zhang, Miao Liu, Duo-Quan Wang, Sheng-Qun Deng

**Affiliations:** 1 The Key Laboratory of Microbiology and Parasitology of Anhui Province, the Key Laboratory of Zoonoses of High Institutions in Anhui, Department of Pathogen Biology, School of Basic Medical Sciences, Anhui Medical University, Hefei, China; 2 Department of Radiotherapy, The First Affiliated Hospital of Anhui Medical University, Hefei, China; 3 Chinese Center for Disease Control and Prevention, National Institute of Parasitic Diseases, Shanghai, China

**Keywords:** Sterile insect technique, Heptacosane, Male mating competitiveness, *Aedes aegypti*, X-ray, γ-ray

## Abstract

**Background:**

*Aedes aegypti* is a vector that transmits various viral diseases, including dengue and Zika. The radiation-based sterile insect technique (SIT) has a limited effect on mosquito control because of the difficulty in irradiating males without reducing their mating competitiveness. In this study, the insect sex pheromone heptacosane was applied to *Ae. aegypti* males to investigate whether it could enhance the mating competitiveness of irradiated males.

**Methods:**

Heptacosane was smeared on the abdomens of *Ae. aegypti* males that were allowed to mate with untreated virgin females. The insemination rate was used to assess the attractiveness of heptacosane-treated males to females. The pupae were irradiated with different doses of X-rays and γ-rays, and the emergence, survival time, egg number, and hatch rate were detected to find the optimal dose of X-ray and γ-ray radiation. The males irradiated at the optimal dose were smeared with heptacosane, released in different ratios with untreated males, and mated with females. The effect of heptacosane on the mating competitiveness of irradiated mosquitoes was then evaluated by the hatch rate, induced sterility, and mating competitiveness index.

**Results:**

Applying heptacosane to *Ae. aegypti* males significantly increased the insemination rate of females by 20%. Pupal radiation did not affect egg number but significantly reduced survival time and hatch rate. The emergence of the pupae was not affected by X-ray radiation but was affected by γ-ray radiation. Pupae exposed to 60 Gy X-rays and 40 Gy γ-rays were selected for subsequent experiments. After 60 Gy X-ray irradiation or 40 Gy γ-ray irradiation, the average hatch rate was less than 0.1%, and the average survival time was more than 15 days. Moreover, at the same release ratio, the hatch rate of the irradiated group perfumed with heptacosane was lower than that of the group without heptacosane. Conversely, the male sterility and male mating competitiveness index were significantly increased due to the use of heptacosane.

**Conclusions:**

The sex pheromone heptacosane enhanced the interaction between *Ae. aegypti* males and females. Perfuming males irradiated by X-rays or γ-rays with heptacosane led to a significant increase in mating competitiveness. This study provided a new idea for improving the application effect of SIT.

**Graphical Abstract:**

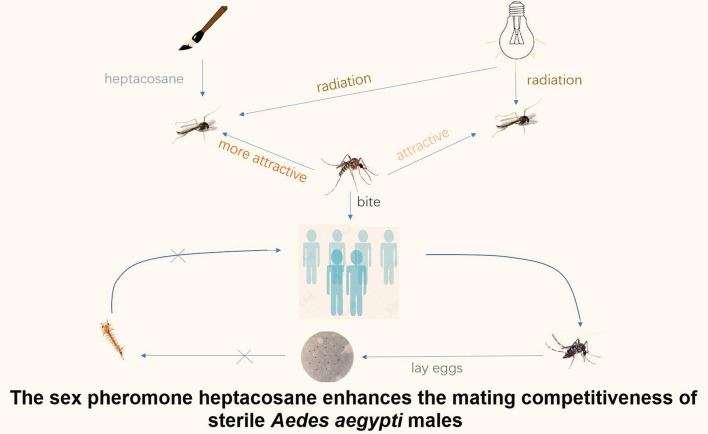

## Background

*Aedes aegypti* is a vector responsible for transmitting various viral diseases, including dengue, Zika, chikungunya, and yellow fever, which pose a great threat to human health [1]. The use of chemical insecticides to control mosquitoes remains the most effective means of combating these mosquito-borne diseases [2]. However, the extensive use of chemical pesticides can make mosquitoes resistant to pesticides, and pesticide residues pose a threat to humans and non-target organisms [3, 4]. Thus, the need to find new mosquito control methods is urgent.

The sterile insect technique (SIT) involves releasing a large number of irradiated sterile male mosquitoes to compete with wild males for female mates to suppress the mosquito population [5]. This technique is species-specific, environmentally friendly, and suitable for large-scale control [6]. For better control of disease-transmitting mosquitoes, the Food and Agriculture Organization of the United Nations and the International Atomic Energy Agency have increased efforts to comprehensively develop and improve SIT packages for the area-wide management of mosquitoes to support their member states [7]. Several countries are currently conducting pilot trials to evaluate the efficacy of SIT. For example, the National Institute of Public Health in Mexico is evaluating the possibility of SIT as an additional control measure for local *Ae. aegypti* and *Ae. albopictus* in the most affected areas of the country [8]. Furthermore, feasibility studies on the use of SIT against invasive *Ae. albopictus* started in 2000, including several field tests on the release of irradiated males [9, 10]. In addition, radiation-based sterile insect techniques were combined with *Wolbachia*-induced insect incompatibility techniques to control mosquitoes in the field (known as insect incompatibility technique/SIT [IIT-SIT]) [5]. After sex separation and radiation, any female with *Wolbachia* accidentally released would not be able to reproduce, thus minimizing any unwanted establishment of *Wolbachia* in the wild. The use of IIT-SIT has successfully controlled *Ae. albopictus* in field trials in China [5] and suppressed the natural population of *Ae. aegypti* in Singapore [11], Thailand [12], and Mexico [13].

Gamma rays are the most commonly used radiation source in SIT, with high photon energy and strong penetrating ability, but with the stricter management of radioactive substances in most countries, the acquisition of γ-ray sources has become increasingly difficult. In contrast, X-ray equipment is easier to obtain, simpler to operate, and safer, but its penetrating power is weaker than that of γ-rays [14]. After male mosquitoes are irradiated by X-rays or γ-rays, their survival time and ability to compete with unirradiated male mosquitoes for mating with females are significantly affected, which also dramatically limits the wide application of this technology [15].

Pheromones are chemical signals used in communication between individuals of the same species, and their roles include attraction, aggression, aphrodisiac, anti-aphrodisiac, aggregation, kin recognition, and alarm signal recognition [16, 17]. In many Diptera insect species, such as housefly, fruit fly, vinegar fly, and mosquito, long-chain diolefins and mono-olefins (cuticular hydrocarbons, CHCs) found on the surface of the epidermis act as attractants and aphrodisiacs, affecting mate choice and inducing courtship [18]. The CHCs tricosane and heptacosane have been isolated and identified from epidermal extracts of mature males of *Anopheles stephensi,* and it was demonstrated that heptacosane enhances the attractiveness of sexually mature males to courtship females [19]. However, little is known about these pheromones in *Aedes* mosquitoes. Therefore, we wondered whether the sex pheromone of *Anopheles* mosquitoes affects the mating activity of *Aedes* mosquitoes. In this study, we compared the effects of CHC components (tricosane and heptacosane) on the mating success of male adult *Ae. aegypti* mosquitoes. In addition, we also combined pheromone with SIT (X-ray and γ-ray irradiation of male mosquitoes) to explore whether pheromone can enhance the competitiveness of sterile male mosquitoes and achieve a better inhibitory effect on mosquito populations.

## Methods

### Mosquito

*Aedes aegypti* mosquitoes from Zhanjiang City, Guangdong Province, China, were collected by the Guangdong Provincial Center for Disease Control and Prevention. This colony was maintained under conditions of 28 ± 1 °C, 80 ± 5% relative humidity, and a light/dark cycle of 16 h/8 h. The mosquito larvae were fed daily with turtle food, and adults were provided with 10% glucose solution ad libitum. Kunming mouse blood (provided by the Animal Experiment Center of Anhui Medical University) was allowed to feed adult females to lay eggs.

### Radiation instruments

The Varian Clinac 23EX linear accelerator (Varian, Palo Alto, CA, USA) and Biobeam GM2000 γRay irradiation device (Gamma-Service Medical GmbH, Leipzig, Germany) were used for X- and γ-ray radiation, respectively.

###  Effect of heptacosane and tricosane on the mating activity of *Ae. aegypti*

Heptacosane and tricosane were dissolved in *n*-hexane at a concentration of 75 µg/ml and applied to the abdomen of 2-day-old *Ae. aegypti* males using paintbrushes as described by Wang et al. [19]. The solvent *n*-hexane was used as a control. Forty-eight hours later, 20 treated males and 20 virgin females were introduced into a cage (25 × 35 × 25 cm) and allowed to mate overnight. Then, the female spermathecae were dissected, and the insemination status was examined. When sperm was detected in at least one of the three spermathecae, the mosquito was considered successfully inseminated. Each treatment was replicated three times, with 20 mosquitoes per replicate, and the mating activity tests were repeated three times.

### Effects of pupal irradiation on emergence, survival, egg number, and hatch rate

Depending on the size and color of the pupae, smaller and lighter *Ae. aegypti* male pupae were selected. Then, the selected male pupae (12–24 h old, 150–200/tray) were placed in the center of the tray (diameter: 9 cm). The excess water in the tray was removed with a straw. The tray was placed at the bottom of the X-ray or γ-ray irradiation chamber. Mosquito pupae were exposed to X-rays or γ-rays at a dose rate of 200 Gy/h. After 20 Gy, 40 Gy, or 60 Gy irradiation, pupae were transferred to clean cages (25 × 35 × 25 cm). After 48 h, the non-emerged dead pupae were counted to evaluate the emergence rate. Moreover, 30 unirradiated and virgin females were added to each cage (holding 30 irradiated males) for mating overnight and then blood-feeding. Blood-fed females were placed in individual 70-ml tubes containing wet filter papers for oviposition. The females were fed Kunming mouse blood only once, and the same was true for collecting eggs. After 5 days, the filter papers holding the eggs were left to dry for 24 h under ambient conditions and then placed in a water basin for 7 days for hatching. Female fertility was calculated by recording the individual numbers of eggs under the microscope and the hatch rates. In addition, pupae exposed to 20 Gy, 40 Gy, and 60 Gy were chosen to examine the effects of X-ray or γ-ray radiation on the survival time of male adults. Three replicates of 30 pupae or adult mosquitoes were used in each treatment, including the controls (unirradiated groups). Males were fed a 10% glucose solution.

###  Effect of heptacosane on the mating competitiveness of irradiated mosquitoes

In combination with the above experimental results (relatively little impact on the survival time of males and a better effect on reducing the hatch rate), pupae exposed to 60 Gy X-rays and 40 Gy γ-rays were chosen to examine the effects of heptacosane (while tricosane did not significantly enhance the insemination rate) on the male mating competitiveness of irradiated mosquitoes.

Male pupae (12–24 h old) were irradiated with 60 Gy X-ray or 40 Gy γ-ray and then transferred to clean cages for emergence. After 48 h, heptacosane was smeared on the abdomen of the males. Then, 30, 30, 90, 150, and 210 treated males were moved to different cages containing 0, 30, 30, 30, and 30 unirradiated males. The release ratios [(irradiated + heptacosane)/unirradiated; I-H/U] were 1:0, 1:1, 3:1, 5:1, and 7:1. Irradiated males without heptacosane and unirradiated male mosquitoes were released in the same release ratios (irradiated/unirradiated; I/U) as controls. After 24 h, 30 female mosquitoes (unirradiated virgins, 5–7 days old) were placed in each cage for mating competition for 3 days. Then, females were fed Kunming mouse blood. After 5 days, eggs were collected and allowed to hatch for 7 days. The hatch rate for each group was recorded. Three replicates were performed for each group. The induced sterility (IS) and the male mating competitiveness index (*C*) were calculated according to the following formulas [20, 21]:

$${\text{IS}}\, = \,(\left( {{1}{-}{\text{ Hc}}/{\text{Hn}}} \right)\, \times \,{1}00\%$$

$$C\, = \,\left( {\left( {{\text{Hn}} - {\text{Hc}}} \right)/\left( {{\text{Hc}} - {\text{Hs}}} \right)} \right)\, \times \,({\text{N}}/{\text{S}})$$

where Hs is the hatch rate of the irradiated control group, Hc is the hatch rate of the competition group (a mixed ratio of unirradiated and irradiated males), Hn is the hatch rate of the unirradiated control group, N is the number of unirradiated males, and S is the number of irradiated males.

### Statistical analysis

Statistical analyses were performed using IBM SPSS version 20. Differences in emergence and hatch rates among groups were compared using Pearson's Chi-square test and Bonferroni test. Analysis of variance (ANOVA) and Tukey's post hoc test were used to compare differences in egg number, IS, and *C* between groups. Kaplan‒Meier analysis was performed to determine relative differences in survival time among groups. Values of *P* < 0.05 were considered statistically significant.

## Results

### Effect of heptacosane and tricosane on the mating activity of *Aedes* mosquitoes

The average insemination rate of female *Ae. aegypti* mosquitoes in the heptacosane-treated group was 60.6 ± 1.6%, which was significantly higher than the 36.1 ± 2.3% in the tricosane group (χ^2^ = 21.535, df = 1, *P* < 0.001) and 40.6 ± 2.5% in the *n*-hexane control group (χ^2^ = 14.402, df = 1, *P* < 0.001). However, there was no significant difference in the insemination rate of *Ae. aegypti* females between the *n*-hexane group and the tricosane control group (χ^2^ = 0.752, df = 1, *P* = 0.386). Therefore, applying tricosane to mature male *Ae. aegypti* mosquitoes did not affect their mating activities, while applying heptacosane significantly increased the insemination rate of *Ae. aegypti* females by 20%.

### Effects of pupal irradiation on emergence, egg number, and hatch rate

There was no effect of radiation dose on the number of eggs laid per female, regardless of the kind of used radiation ray (χ^2^ = 6.584, df = 3, *P* = 0.086) (Table 1). However, when the γ-ray radiation dose reached 40 Gy or 60 Gy, the pupal emergence rate was significantly lower than that of the control group (χ^2^ = 39.155, df = 3, *P* < 0.001) (Table 1).

**Table 1:** Effects of pupal irradiation on emergence, egg number, and hatch rate

Irradiation	Emergence rate (%)	Egg number	Hatch rate (%)
Control	92.6 ± 3.2 a	66.6 ± 1.9 a	85.9 ± 6.6 a
X-ray 20 Gy	90.0 ± 4.1 a, b	65.5 ± 2.2 a	29.2 ± 4.2 b
X-ray 40 Gy	87.4 ± 3.2 a, b	63.5 ± 2.5 a	5.2 ± 2.5 c
X-ray 60 Gy	86.3 ± 3.5 a, b	65.0 ± 2.3 a	0.3 ± 0.5 d
γ-ray 20 Gy	88.9 ± 2.4 a, b	68.1 ± 2.2 a	6.0 ± 1.8 c
γ-ray 40 Gy	83.4 ± 3.5 b, c	68.0 ± 2.3 a	0.2 ± 0.4 d
γ-ray 60 Gy	74.4 ± 4.4 c	64.8 ± 2.2 a	0 ± 0 d

Data are presented as the mean ± SE. Values followed by different letters are significantly different from each other (emergence rate and hatch rate: Pearson's Chi-square test and Bonferroni test, *P* < 0.05; egg number: ANOVA and Tukey's post hoc test)

**Fig. 1:**
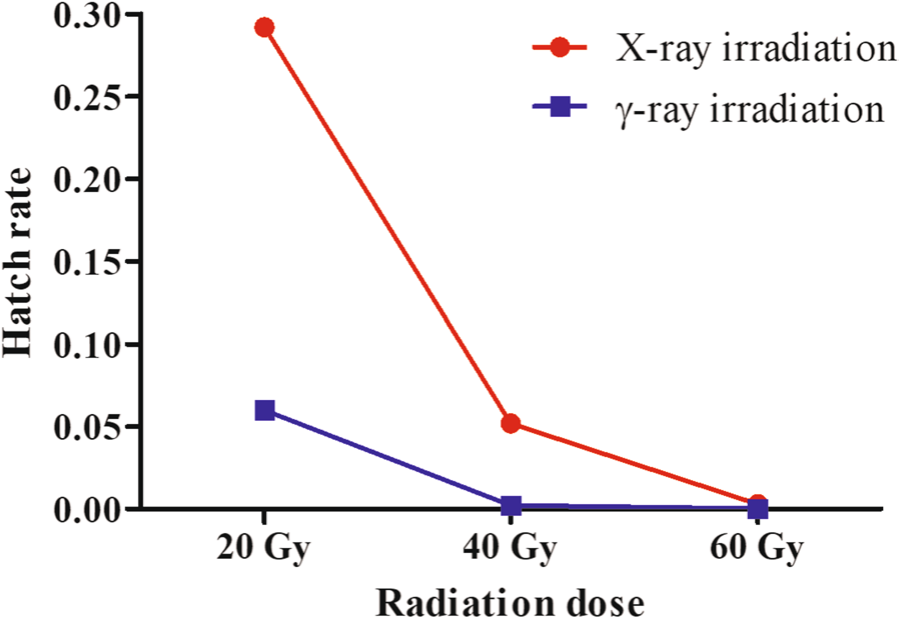
Effects of pupal irradiation on the hatch rate

Moreover, male pupae radiation (including X-ray and γ-ray radiation) was not correlated with female egg number (F = 0.592, df = 6, *P* = 0.737). In contrast, the hatch rate decreased significantly with increasing radiation dose (χ^2^ = 3635.000, df = 6, *P* < 0.001) (Fig. 1). After 60 Gy X-ray or more than or equal to 40 Gy γ-ray radiation, the hatch rate was less than 0.1%. Furthermore, no egg was hatched after 60 Gy γ-ray irradiation (Table 1).

### Effect of pupal radiation on the survival of male mosquitoes

**Fig. 2:**
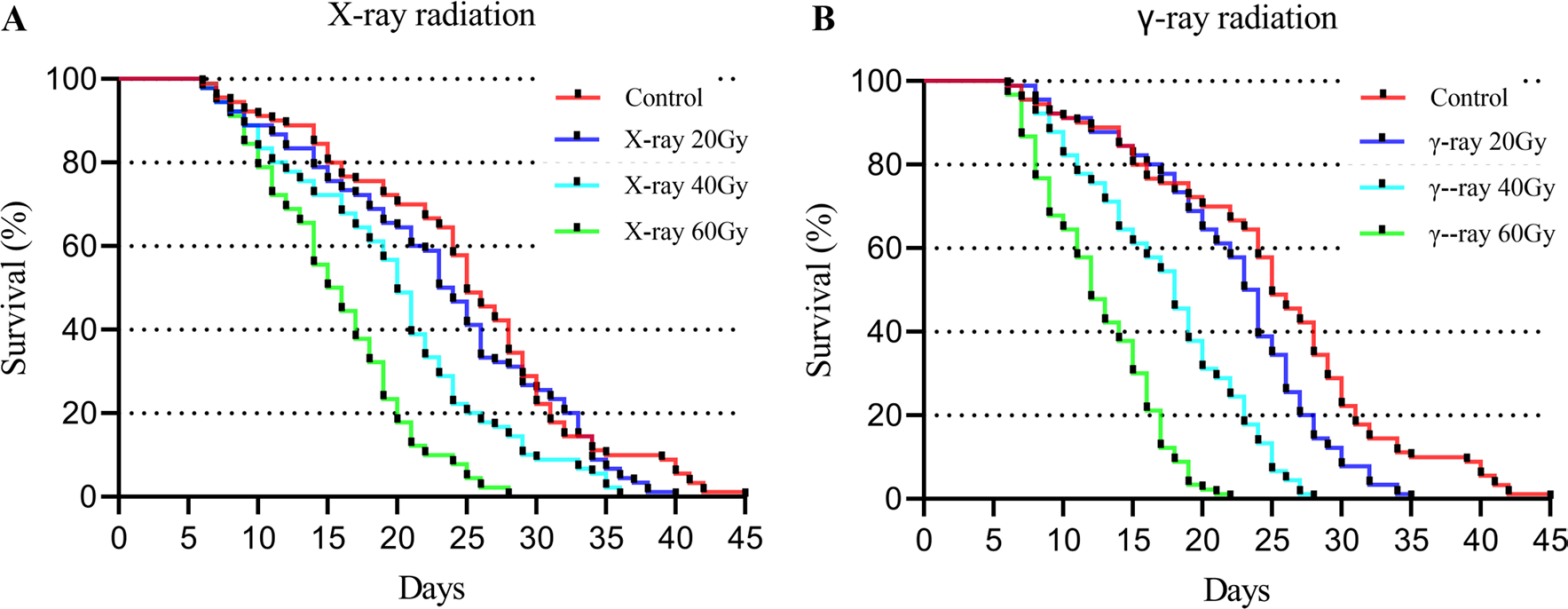
Survival curves of *Aedes aegypti* males irradiated with different doses of X-rays (**A**) and γ-rays (**B**)

The average survival time of the males in the control groups was 24.7 ± 1.0 days. At the same time, the average survival times of adult males were 23.0 ± 0.9, 19.8 ± 0.8, and 15.6 ± 0.6 days after exposure to 20 Gy, 40 Gy, and 60 Gy X-rays, respectively (Table 2). The survival of male mosquitoes tended to decrease with increasing X-ray radiation dose. Compared with the control group, 40 Gy and 60 Gy X-ray irradiation of pupae significantly reduced the survival time of adult male mosquitoes (Fig. 2A). However, no significant difference in male longevity was observed between 20 Gy X-ray irradiation of pupae and the control group. In addition, the average survival times of male adults were 22.1 ± 0.7, 17.3 ± 0.6, and 12.6 ± 0.4 days after exposure to 20 Gy, 40 Gy, and 60 Gy γ-rays, respectively. Here, again, the survival time was inversely proportional to the radiation dose (Fig. 2B). The survival times for all γ-ray irradiation doses were significantly lower than that of the control.

**Table 2:** Egg hatch rates at different release ratios

Treatment	Release ratio	Hatch rate (X-ray, %)	Hatch rate (γ-ray, %)
Control	0:1	84.2 ± 4.1 a	86.3 ± 4.0 a
I/U	1:1	55.4 ± 9.4 b	61.1 ± 4.4 b
	3:1	36.0 ± 8.0 c	40.6 ± 5.0 c
	5:1	30.4 ± 3.5 c, d	28.3 ± 5.5 d, e
	7:1	23.9 ± 5.5 d, e	21.7 ± 4.8 e, f
	1:0	0.7 ± 1.1 f	0.4 ± 0.7 g
I-H/U	1:1	46.1 ± 7.6 g	49.2 ± 8.3 h
	3:1	31.1 ± 6.2 c	34.6 ± 5.8 c, d
	5:1	22.9 ± 6.8 e, h	21.0 ± 5.0 f
	7:1	17.2 ± 2.6 h	16.1 ± 3.4 f
	1:0	0.6 ± 0.9 f	0.3 ± 0.7 g

*I/U* irradiated/unirradiated; *I-H/U* (irradiated + heptacosane)/unirradiated. Data are presented as the mean ± SE. Values followed by different letters are significantly different from each other (Pearson's Chi-square test and Bonferroni test, *P* < 0.05)

###  Effect of heptacosane on the mating competitiveness of irradiated mosquitoes

Pupae exposed to 60 Gy X-rays and 40 Gy γ-rays were used to study the effect of heptacosane on the mating competitiveness of irradiated mosquito males. After mating with irradiated males, the hatch rate of wild females decreased with the increase in release ratio, and the difference was significant (X-ray irradiation: χ^2^ = 1556.853, df = 5, *P* < 0.001; γ-ray irradiation: χ^2^ = 1739.767, df = 5, *P* < 0.001). These trends were also observed in experiments releasing heptacosane-coated male mosquitoes (X-ray irradiation: χ^2^ = 1692.237, df = 5, *P* < 0.001; γ-ray irradiation: χ^2^ = 1826.224, df = 5, *P* < 0.001). Moreover, the hatch rate of the group coated with heptacosane was lower than that of the group without heptacosane at the same release ratio. When the release ratios were 1:1, 5:1, and 7:1 in the X-ray radiation experiment and 1:1 and 5:1 in the γ-ray radiation experiment, the difference was statistically significant (Table 2).

**Fig. 3:**
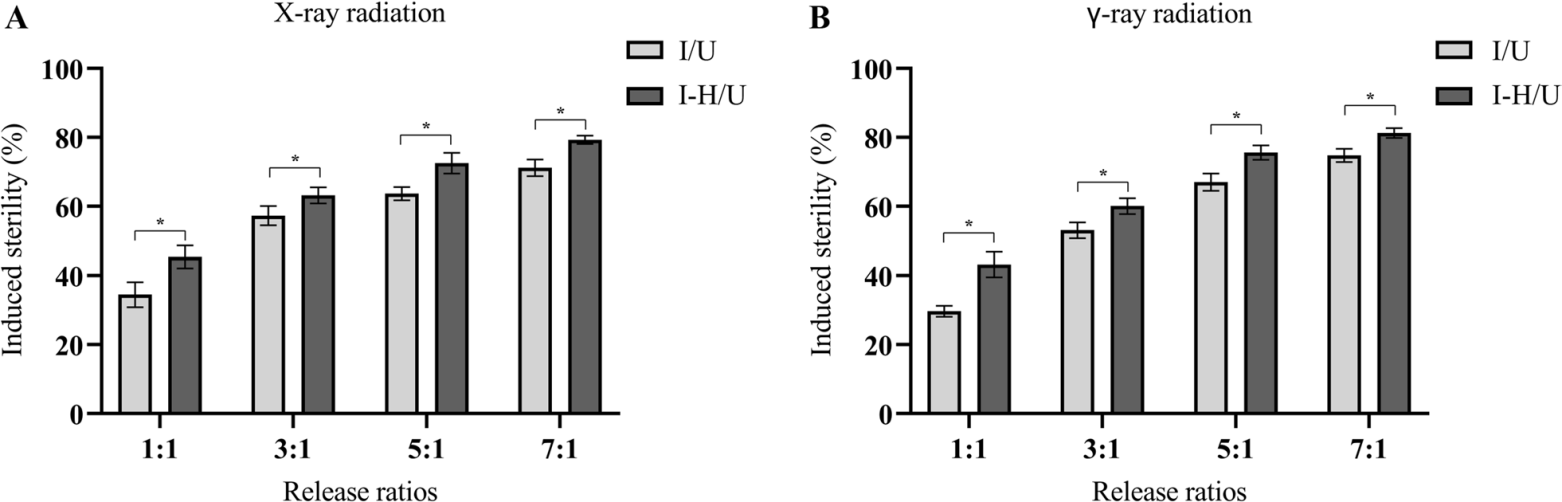
Effects of heptacosane on induced sterility of *Aedes aegypti* irradiated by X-rays (**A**) and γ-rays (**B**). The error bar indicates ± SE. *P < 0.05 (ANOVA and Tukey's post hoc test)

**Fig. 4:**
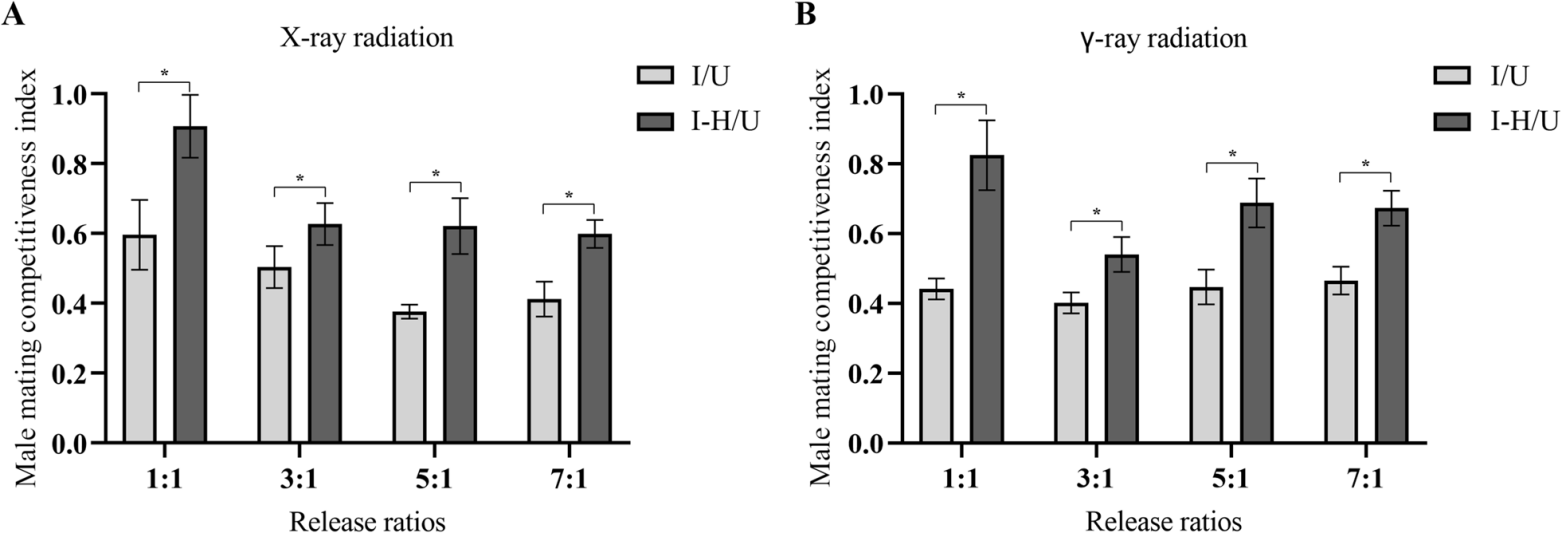
Effects of heptacosane on the male mating competitiveness index of *Aedes aegypti* irradiated by X-rays (**A**) and γ-rays (**B**). The error bar indicates ± SE. *P < 0.05 (ANOVA and Tukey's post hoc test)

 Furthermore, the induced sterility increased with the increase in the release ratio, and when at the same release ratio, the induced sterility of the male mosquitoes perfused with heptacosane was significantly higher than that of the untreated group (Fig. 3). Similarly, at the same release ratio, the mating competitiveness index (*C*) of male mosquitoes perfumed with heptacosane was significantly higher than that of male mosquitoes not perfumed with heptacosane (Fig. 4).

## Discussion

*Aedes aegypti* is a highly efficient vector of the dengue virus and Zika virus [22]. It likes to go out in the early morning and the evening before dusk to find the host to suck blood [7]. This means that insecticide-treated mosquito nets are ineffective in preventing dengue and Zika transmission, unlike malaria. SIT, a concept proposed by Edward Knipling in the 1950s, is a species-specific, pollution-free, and eco-friendly method for pest control [23, 24]. Radiation-based SIT has been successfully used to control mosquitoes and reduce the incidence of vector-borne diseases [25]. Most laboratory studies and field trials have used γ-rays, and X-rays were rarely used to irradiate mosquitoes [26–28]. In our study, *Ae. aegypti* pupae were irradiated with X-rays and γ-rays, and the differences in emergence, survival time, egg number, and hatch rate of the two types of radiation were compared.

The emergence of the adults was not affected by X-ray radiation but was affected by γ-ray radiation. The additional deaths may be due to the greater penetration of γ-rays, which destroy more of the mosquito's somatic cells. Similar findings were reported for *Ae. albopictus*. X-rays did not affect the emergence rate of *Ae. albopictus*, but γ-rays significantly reduced the emergence rate of *Ae. albopictus* [29, 30].

Moreover, in the present study, pupal radiation did not affect the egg number of females but significantly reduced the hatch rate, especially when γ-ray radiation was used. For example, 40 Gy γ-ray radiation had the same effect on the hatch rate as 60 Gy X-ray radiation. The destructive ability of γ-rays on mosquito sperm cells was stronger than that of X-rays. Similarly, the survival time of male mosquitoes that emerged after pupae irradiation was significantly reduced. The effect of γ-rays on the survival of male mosquitoes is higher than that of X-rays at the same doses. Moreover, Chen et al. and Shetty et al. irradiated *Ae. aegypti* with different doses of γ-rays. They also found that high-dose radiation did not affect the fecundity of females but had a negative impact on the survival time and mating competitiveness of irradiated male mosquitoes [31, 32]. Similarly, Rodriguez et al. proved that X-ray radiation did not affect egg numbers, but the survival time of *Ae. aegypti* males decreased with increasing radiation dose [33]. A study by Yamada et al. showed that X-rays generated by Raycell Mk2 irradiators induced comparable sterility levels for *Ae. aegypti* males compared to γ-rays [34]. In addition, in our previous study, we used different doses of X-rays and γ-rays to irradiate *Ae. albopictus* and obtained similar results as *Ae. aegypti* [35]. Based on the above results, although male mosquitoes could be sterile by radiation, the mating ability of the irradiated males was significantly affected, which greatly reduced the application effect of SIT.

Chemical pheromones play an important role in female mosquitoes' search for, identification of, and selection of mating males [36]. For example, the pheromone heptacosane (a cuticular hydrocarbon) enhanced the interaction between *Anopheles* males and females [19]. Heptacosane is also a contact and volatile pheromone that promotes the mating activity of the tea weevil *Myllocerinus aurolineatus* [37]. In addition, heptacosane was one of the main components of pheromone in the termite *Reticulitermes speratus*, which could induce long-term aggregation at new nesting and feeding sites [38]. In the present study, applying heptacosane to mature *Ae. aegypti* males significantly increased the insemination rate of females. Therefore, we used this sex pheromone in combination with SIT, hoping to enhance the population-suppressing effect of releasing irradiated male mosquitoes. Surprisingly, we found that the induced sterility and mating competitiveness index of the male mosquitoes smeared with heptacosane was significantly higher than that of the non-smeared group. When the release ratio of irradiated male mosquitoes smeared with heptacosane to normal male mosquitoes was 5:1, the sterility effect was equivalent to that of the non-smeared group at a release ratio of 7:1. These results suggested that perfuming heptacosane to sterile mosquitoes can enhance the inhibitory effect on the mosquito population with the same release amount. In addition, field releases of *Wolbachia*-infected *Ae. aegypti* are being implemented in multiple countries. Insect sex pheromones heptacosane may also be used in IIT or IIT-SIT in the future to enhance the population inhibition effect of sterile mosquitoes released in the field.

However, although our results demonstrate the good effect of SIT in combination with heptacosane at the laboratory level, there are still many problems to be solved before the actual application of mosquito control in the field. First, heptacosane has strong volatility, and how can its continuous effect on releasing males be ensured? Sun et al. reported that 10 μg/ml heptacosane solution applied to females of *Myllocerinus aurolineatus* could attract males for mating within 12.04 h on average [34]. Heptacosane may lose its effect on enhancing mating competitiveness after 12 h. It may be necessary to explore suitable formulations for the slow release and sustained action of heptacosane. Moreover, Fawaz et al. reported that excitation and attraction were observed when *Ae. aegypti* females were exposed to the pheromones 2,6,6-trimethylcyclohex-2-ene-1,4-dione, 2,2,6 trimethylcyclohexane-1,4-dione, or 1-(4-ethylphenyl) ethanone [39]. Perhaps in the future, we can find the long-range male sex pheromones that females use to find and identify males to attract females to actively mate with sterile males in the field. A recent study by Mozöraitis et al. highlighted the presence of five volatile compounds (acetoin, sulcatone, octanal, nonanal, and decanal) that *Anopheles* males released. According to the authors, these might be related to aggregation behaviors that attract males and females and increase the insemination rate [40]. Nevertheless, a laboratory study failed to demonstrate the long-range male sex pheromones associated with swarm detection and recognition by females [41]. They repeated the protocol by Mozūraitis et al. and found that acetoin was absent from almost all the male samples. The other four compounds (sulcatone, octanal, nonanal, and decanal) were detected but did not differ significantly from the control group [41]. The high quantitative variability found in the laboratory and the fact that these compounds are often found in the control indicate that they may be uncontrollable laboratory and/or human pollution. Overall, our study provides a new idea for applying sterile insect technology in the future. The combination of insect sex pheromones and irradiated mosquitoes may enhance the effect of sterile insects in the field.

## Conclusion

In conclusion, we confirmed that the sex pheromone heptacosane perfumed on the abdomen of *Aedes aegypti* male mosquitoes could enhance their attractiveness to female mosquitoes and thus increase their insemination rate. In addition, when male mosquitoes irradiated by X-rays or γ-rays were smeared with heptacosane, the mating competitiveness of the irradiated male mosquitoes could be significantly enhanced. Although more evidence is needed for applying the sex pheromone in SIT in the field, our experiments undoubtedly provide new ideas for the broader application of SIT.

## Data Availability

All data generated or analyzed during this study are included in this published article.

## Abbreviations

SIT: Sterile insect technique
IIT: Insect incompatibility technique
CHC: Cuticular hydrocarbon
I-H/U: (Irradiated + heptacosane)/unirradiated
I/U: Irradiated/unirradiated
IS: Induced sterility
*C*: Mating competitiveness index
