# Correction: The sex pheromone heptacosane enhances the mating competitiveness of sterile *Aedes aegypti* males

**DOI:** 10.1186/s13071-023-06078-4

**Published:** 2023-12-11

**Authors:** Lin‑Min Wang, Ni Li, Mao Zhang, Qi Tang, Hong‑Zheng Lu, Qing‑Ya Zhou, Jia‑Xuan Niu, Liang Xiao, Zhe‑Yu Peng, Chao Zhang, Miao Liu, Duo‑Quan Wang, Sheng‑Qun Deng

**Affiliations:** 1 The Key Laboratory of Microbiology and Parasitology of Anhui Province, The Key Laboratory of Zoonoses of High Institutions in Anhui, Department of Pathogen Biology, School of Basic Medical Sciences, Anhui Medical University, Hefei, China; 2 Department of Radiotherapy, The First Affiliated Hospital of Anhui Medical University, Hefei, China; 3 Chinese Center for Disease Control and Prevention, National Institute of Parasitic Diseases, Shanghai, China

**Correction: Parasites & Vectors (2023) 16:102** **https://doi.org/10.1186/s13071-023-05711-6**

Following publication of the original article [1], two errors came to the attention of the authors: Mao Zhang had not been indicated as a co-first author, and in the Funding declaration, the funding number of the National Natural Science Foundation of China had been incorrectly detailed as ‘8210082025’ instead of ‘82102432’, the correct number.

These errors have now been rectified in the original article. The authors thank you for reading and apologize for any inconvenience caused.
